# Effects of COVID-19 on Telework and Commuting Behavior: Evidence from 3 Years of Panel Data

**DOI:** 10.1177/03611981221089938

**Published:** 2022-05-07

**Authors:** Anna Reiffer, Miriam Magdolen, Lisa Ecke, Peter Vortisch

**Affiliations:** 1 Institute for Transport Studies, Karlsruhe Institute of Technology, Karlsruhe, Germany

**Keywords:** planning and analysis, effects of information and communication technologies (ICT) on travel choices, telecommuting, traveler behavior and values, behavior analysis, pattern (behavior, choices, etc.)

## Abstract

The COVID-19 pandemic has forced employers and employees to re-evaluate their attitudes toward telecommuting. This induced a change in the sheer number of people who have started to work from home (WFH). While previous studies highlight differences between telecommuters based on their level of telecommuting experience, these effects have not been studied in detail. This may limit the evaluation of implications for post-pandemic times and the transferability of models and predictions based on data collected during the COVID-19 pandemic. This study expands on previous findings by comparing the characteristics and behavior of those who have started to telecommute during the pandemic and those who had already telecommuted before. Furthermore, this study addresses the uncertainty that exists about whether the findings of studies conducted before the pandemic—for example about sociodemographic characteristics of telecommuters—still hold true, or if the pandemic induced a shift in telecommuters’ profiles. Telecommuters show differences when considering their previous experience in WFH. The results of this study suggest that the transition induced by the pandemic was more drastic for new telecommuters compared with experienced telecommuters. The COVID-19 pandemic had an effect on how household configurations are considered in the choice to WFH. With decreased access to child care resulting from school closings, people with children in the household were more likely to choose to telecommute during the pandemic. Also, while people living alone are generally less likely to choose to WFH, this effect was reduced as a result of the pandemic.

The outbreak of the Coronavirus disease in 2019 (COVID-19) in Wuhan, China, and the subsequent declaration of a pandemic had a significant impact on people’s lives and behavior (*1*). In an attempt to limit the spread of the disease, many governments urged their citizens to practice social distancing and mandated preventive measures. Albeit at different times and with varying intensity levels, most countries resorted to similar policies: mandates to wear a mask in public, stay-at-home requirements, social distancing, and the closing of shops, restaurants, schools, and workplaces.

Significant changes in mobility patterns were observed, arising from the changes in activity levels and perceptions of safety. Several studies have reported modal shifts and an overall reduction of traffic. While some of these changes in travel behavior seem disconcerting because of the shift toward less environmentally friendly modes, once mandates are revoked and the spread of the disease has reached a tolerable level, it can be expected that most of these changes will return to the pre-pandemic state (*2*–*4*). Results presented by Molloy et al. indicate that these changes regress with increased relaxation of policy measures (*3*). Opposed to this are possible permanent changes in (travel) behavior such as telecommuting patterns. In the context of this study, telecommuting describes the act of working from home while utilizing information and communication technologies (ICT). Recent evidence suggests that employees who were allowed to work from home (WFH) during the pandemic will want to continue to have this opportunity after the pandemic. Research on possible telecommuting effects was first conducted in the 1970s (*4*). Since then, a considerable amount of literature has been published on these effects, trying to settle the debate on whether telecommuting alleviates transport problems or exacerbates them. While some studies suggest that not only commuting trips are reduced through telecommuting but also the total number of trips and vehicle miles traveled (VMT), other studies present contradictory findings especially because of rebound effects and residential relocation (*5* [Bibr bibr6-03611981221089938][Bibr bibr7-03611981221089938][Bibr bibr8-03611981221089938][Bibr bibr9-03611981221089938][Bibr bibr10-03611981221089938][Bibr bibr11-03611981221089938][Bibr bibr12-03611981221089938][Bibr bibr13-03611981221089938][Bibr bibr14-03611981221089938][Bibr bibr15-03611981221089938]–*16*). Although this is an important debate, the impact of the discussed effects had been relatively low because of the low adoption rate of telecommuting before the pandemic. Projection of telework rates from 1999 to 2005 for European countries estimated that home-based teleworkers would make up 4% to 7% of the German labor force (*17*). However, only 1.4% of respondents of a survey conducted in 2005 stated that they are regularly involved in telework (*18*). Ten years later, the numbers were still not as high as expected, with less than 2% of workers in Germany in 2015 being home-based teleworkers (*19*). The slow adoption can be attributed to three factors. Firstly, not every job can be conducted remotely, and telework has been a privilege mainly granted to highly educated professionals and managers (*18, 20–22*). Secondly, employers have to allow their employees to work outside the usual workplace, which was highly dependent on the attitude of managers toward telework before the pandemic (*23*). And, thirdly, employees also have to choose to work remotely. While telework may be linked to a better work-life balance, higher productivity because of fewer distractions, and overall higher job satisfaction, some employees like going to work on-site for social interaction or because they feel the need to separate their workplace from their personal space (*24*, *25*). Because there are various factors influencing the possibility and the choice to telecommute, it has to be investigated if there have been changes in the effect of these factors on telework and travel behavior induced by the COVID-19 pandemic.

This paper investigates shifts induced by the pandemic on the possibility to telecommute and employees’ decision to do so, based on descriptive analyses and regression models. Furthermore, this study explores differences between non-telecommuters, experienced telecommuters, and new telecommuters. To investigate the effect the COVID-19 pandemic had on telecommuting, data from 3 years of the German Mobility Panel (MOP)—a longitudinal national household travel survey—was analyzed. By using data from cohorts that participated not only during the pandemic but also in 2018 and 2019, it is possible to capture changes in behavior from the same individuals.

The paper is structured as follows. First, a concise overview of studies into the effects of the COVID-19 pandemic on telecommuting and travel behavior is provided. In the subsequent section on materials and methods, information is provided on the MOP, the variables used, and the theoretical foundation of the regression models. The results section provides key insights from the survey cohorts that participated in 2018 and/or 2019 and 2020 concerning telecommuting and related travel behavior and the results of the regression models. Finally, the results are discussed and the paper concludes with implications for both pandemic and post-pandemic times.

## Literature

The COVID-19 pandemic forced employers and employees to set aside their attitudes toward WFH, as social interactions during the commute and in the workplace have become associated with the risk of infection. This induced a change in the sheer number of people who started to WFH. The changes in working and commuting behavior have been reported in several studies. Shamshiripour et al. reported that 71% of respondents from the Chicago metropolitan area survey had never done WFH before the pandemic (*26*). Similar findings were presented by de Haas et al. (*27*). Their analysis of the Netherlands Mobility Panel found that 44% of employed respondents have started WFH regularly during the pandemic and 58% attested that WFH is a new experience for them. Beck et al. presented findings from a survey in Australia, showing that the share of respondents who do not engage in WFH dropped from 71% before the pandemic to 39% during a survey wave with relatively high infection rates (*28*). The same survey also indicates that the number of days of WFH increased because of the pandemic: the rate of respondents WFH 5 days a week increased from 7% to 30%. Similar findings are reported by Hiselius and Arnfalk from a survey among employees of Swedish public agencies. A total of 66% of respondents from that survey attested that they stopped commuting all together and 16% only commuted 1 to 2 days a week (*29*).

Considering the long-term effects of the pandemic on attitudes toward WFH, many studies report that people will want to keep WFH in the future. In the Netherlands, de Haas et al. report that over a quarter of respondents expect to keep WFH after the pandemic (*27*). An online survey conducted in Germany shows that around 60% of respondents expect to increase WFH in the future (*30*). Results presented by Beck et al. also show that 71% of respondents would like to WFH more often (*28*). This is in line with the findings that show a high job satisfaction and productivity levels that are at least the same if not higher than before starting to WFH (*26*, *28*, *30*). While these findings imply a positive change toward more people doing WFH and subsequently fewer commuting trips, the pandemic did not change that not everyone can WFH and the possibility to do so seems to remain a privilege for well-educated people earning a high income (*26*, *30*).

A few studies have also highlighted some differences between new and experienced telecommuters and further insights into the effects of the pandemic on telecommuting conditions. Kramer and Kramer show that there are some occupational groups that were able to telework as a result of the COVID-19 pandemic; however, most inequalities identified by earlier studies still hold true (*31*). When comparing new and experienced teleworkers, most studies focus on the difference in perception of telework. Presented studies suggest that new telecommuters positively evaluate telework (*26*, *31*, *32*). However, there are differences when considering telecommuting experience. Hiselius and Arnfalk show that experienced teleworkers are more likely to positively evaluate WFH compared with new teleworkers (*29*). These findings are supported by Shamshiripour et al. (*26*). While the presented results highlight that there are differences between telecommuters based on their level of WFH experience, these effects have not been studied in detail. This may limit the evaluation of implications for post-pandemic times and the transferability of models and predictions based on data collected during the COVID-19 pandemic such as the models presented by Hensher et al. (*33*, *34*).

This study expands on previous findings by comparing characteristics and behavior of those who started to telecommute during the pandemic and those who had already telecommuted before. Furthermore, it is asked if the uncertainty that exists about whether the findings of studies conducted before the pandemic (e.g., about the sociodemographic characteristics of telecommuters) still hold true, or if the pandemic induced a shift in telecommuters’ profiles.

## Materials and Methods

This section first provides an overview of the data that served as a basis for the analyses: MOP. Subsequently, the applied methods are described.

### Data

The analyses in this study are based on data from MOP—a longitudinal national household travel survey that has been conducted annually since 1994. The longitudinal nature of the survey design is twofold. Firstly, respondents participate 3 years in a row and, secondly, they also keep a trip diary for seven consecutive days. Approximately 3,000–3,400 respondents aged 10 years and older in 1,800–2,000 households participate in the survey each year. The survey period is in the fall and excludes any holidays to best capture everyday travel. The trip diary collects information on trip distances, mode of transportation, trip purposes, and start and arrival times. Furthermore, sociodemographic information about the participants (e.g., status of employment, gender, age), and the availability of cars, bicycles, and public transport (PT) passes, as well as certain characteristics of the transportation system facilities (e.g., parking space availability at home and at work), are captured. Moreover, survey participants are asked to report any anomalies such as illness, vacation, and days their car was in the shop. The survey is carried out on behalf of and funded by the German Federal Ministry of Transport and Digital Infrastructure. The Institute for Transport Studies of the Karlsruhe Institute of Technology (KIT) is responsible for the design and scientific supervision of the survey (*35*, *36*).

**Figure 1.**
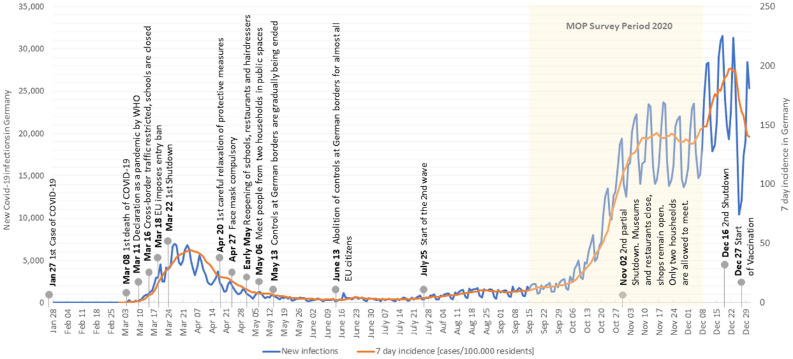
Timeline of COVID-19 cases and implications in 2020 in Germany. *Note*: MOP = German Mobility Panel; WHO = World Health Organization.

The analyses in this study are based on the data from the survey periods 2018 to 2020. The data from 2018 and 2019 serve as pre-COVID-19 reference. The survey period in 2020, and how it relates to new COVID-19 cases and the incidence in Germany, is displayed in Figure 1. The 2019 data were collected before the declaration of the pandemic, whereas the survey of the 2020 period was conducted during the pandemic. During this period, the number of reported infections strongly increased. At the beginning of November, 2020, a second partial lockdown was imposed in Germany, restricting public life. Cultural institutions, such as theatres and museums, were closed, as well as sport facilities. Further, social contacts were reduced to social gatherings of a maximum of 10 people from two different households. However, stores and schools remained open (*37*).

This study is based on a subsample that focuses on employed participants, their travel behavior, and their possibility to WFH. The only respondents who were considered were those aged 18 and older, who were employed on a full-time or part-time basis, and who did not report any anomalies (i.e., illness or vacation), relocation, or job change during the survey period. In total, the subsample contains information from 2,117 reports of 1,138 respondents. The analyses are based around the information on telecommuting reported in the survey in which participants are asked to report their WFH status. They are asked to select one of four categories: “I often work from home” (at least once a week), “I sometimes work from home,”“I have the possibility, but I do not work from home,” and “I do not have the possibility to work from home.”

Table 1 shows the characteristics of the sample used for the analyses. For comparison, the population statistics of 2019 are also provided. It needs to be mentioned that the statistics for the population for some variables, such as occupation status, have been affected considerably by the COVID-19 pandemic. The raw data from MOP show differences to the 2019 statistics. This is because each cohort is recruited to be individually representative of the population. Only those who participate for the second or third time (repeaters) are presented in this study. A study by Chlond et al. shows that young people tend to drop out of the survey prematurely compared with older respondents (*38*). Therefore, young people are underrepresented, whereas older people are overrepresented. In addition, the share of people with a high level of education is large. However, the share of employees between 35 to 50 years is a fairly representative subset. For the analyses, data is weighted by age and gender at the person level to compensate the skewness in the presented data.

**Table 1.** Sample Characteristics

Variable	Level	Raw data (%)	Population^a^ (%)
Gender	Male	48.7	48.8
	Female	51.3	51.2
Age (years)	≤25	0.6	16.9
	26–35	11.3	12.7
	36–50	32.0	19.5
	51–60	40.6	18.5
	≥61	15.5	32.4
Occupation status	Full-time	76.6	70.8
	Part-time	27.4	29.2
Education	Low	9.7	NA
	Medium	28.5	NA
	High	61.8	NA
Economic status (ECS)	Low	8.2	NA
	Medium	45.5	NA
	High	46.3	NA

a Population statistics taken from 2019 (situation before the COVID-19 pandemic) (*39*). NA = not available.

### Methods

To investigate the effects of the COVID-19 pandemic on telecommuting and travel behavior, both behavioral changes over all respondents were analyzed, and also differentiation was made between different telecommuting groups (TGs). For the analysis, three different types of telecommuter were identified:

1. *Experienced telecommuters*: this group includes all employees who indicated that they worked from home frequently or occasionally in 2019 and 2020, respectively.
2. *New telecommuters*: this group includes all respondents who telecommuted only in 2020.
3. *Non-telecommuters*: this group includes all employees of the sample who did not telecommute at all. This includes both respondents who did not have the possibility and those who did have the possibility but chose not to WFH.

For the analyses, both descriptive and inferential methods were applied. Factors that influence the possibility to telecommute and the choice to do so have been assessed using logit models. The effect of the pandemic is analyzed through interaction terms between the independent variables and the year 2020. This makes it possible to identify changes in sociodemographic characteristics of telecommuters induced by the pandemic. To analyze the effects of the pandemic on commuting behavior, a multiple linear regression model has been estimated in which differentiation has also been made between new and experienced telecommuters.

#### Logit Model on the Possibility to Telecommute

To analyze WFH behavior and the influence of the pandemic, several logit models were estimated. All models were estimated using the R package *Apollo*, version 0.2.4 (*40*, *41*). During the estimation process, it became clear that sociodemographic data does not influence if a person *chooses* to WFH but rather if they *can* WFH in the first place. Therefore, a logit model was first estimated to see which personal and household characteristics influence a person’s possibility of WFH. The observations used for this model are from respondents who reported information in 2020 and at least in either 2018 or 2019, which resulted in a dataset of 2,148 observations. As described in the literature, it is mostly male professionals and highly educated people who have the opportunity to WFH. Therefore, variables have been included that describe these circumstances, namely gender, highest degree of education, and the economic status (ECS) of the respondent. To determine the ECS for each respondent, first, the equivalent income is calculated based on the Organization for Economic Co-operation and Development (OECD) square root scale using the household income and size (*42*). The equivalent household income is then partitioned into three parts, where a medium ECS corresponds to an equivalent income between 70% and 150% of the median German income.

To analyze if the COVID-19 pandemic induced a shift in these variables, interaction terms were included. Different specifications of the model were estimated and the shift parameters were tested for all variables; however, only a shift in the alternative specific constant (ASC) and in ECS were statistically significant. The linear predictor *O* used to estimate the model on the opportunity to WFH can be expressed as:

(1) $$\begin{array}{c} O_{n,t}=\mathrm{asc}+\mathrm{as}c_{\mathrm{shift}}\times (t=2020)+\beta_{\mathrm{Gender}}\times \mathrm{Gende}r_{n}\\ +\beta_{\mathrm{edu}}\times \mathrm{Degre}e_{n,t}+(\beta_{\mathrm{ECS}}+\beta_{\mathrm{ECS},\mathrm{shift}}\times \\ \left(t=2020\right))\times \mathrm{EC}S_{n,t} \end{array}$$

where

*asc* = alternative specific constant,

*ECS* = economic status, and

*t* = the survey period of the observation (i.e., 2018, 2019, or 2020).

The probability of person *n* having the possibility to WFH at period *t* is given by:

(2) $$P_{n,t\;}=\;\frac{\exp(O_{n,t})}{1\;+\;\exp(O_{n,t})}$$

As longitudinal data is being used for the estimation with repeated choices for the same person, the probabilities are multiplied across choice observations of the same respondent. The likelihood function is then given by:

(3) $$L_{n\;}=\;\overset{T_{n}}{\underset{t\;=\;1}{\Pi }}P_{n,t\;}$$

#### Logit Model on the Choice to Telecommute

To analyze the pandemic’s effect on the choice of WFH for respondents who can do so, a logit model was also estimated. For the choice model, the answers concerning telecommuting behavior were recoded from the four categories to two: choosing to telecommute and not to telecommute, whereas the answer “I do not have the possibility” was regarded in the previously described model analyzing the possibility to telecommute. To infer which variables influence this choice differently during the COVID-19 pandemic and the state before, shift parameters were again included and interaction was analyzed during different estimation runs. The final model regards the ASC, commuting distance (CD), single households (SHH), couple household (CHH), and children under 10 years old in the household (KiH). CD is only available if the respondent conducted a commuting trip during the survey period. WFH is associated with a lack of commuting trips, thus, this information is missing for several respondents. As CD is still an important explanator, a parameter that accounted for those with missing CDs was also included. Shift parameters were significant for the ASC, SHH, and KiH. The final estimated model can be expressed as:

(4) $$\begin{array}{cc} V_{n,t}= & \mathrm{asc}+\mathrm{as}c_{\mathrm{shift}}\times \left(t=2020\right)+\beta_{\mathrm{commute}}\times CD_{n,t}\times \\ \left(CD_{n,t}>0\right)+\beta_{\mathrm{commute},\mathrm{missing}}\times \left(CD_{n,t}=\mathrm{missing}\right)\\ & +(\beta_{\mathrm{SHH}}+\beta_{\mathrm{SHH},\mathrm{shift}}\times \left(t=2020\right))\times \mathrm{SH}H_{n,t}\\ +\beta_{\mathrm{CHH}}\times \mathrm{CH}H_{n,t}+(\beta_{\mathrm{KiH}}+\beta_{\mathrm{kids},\mathrm{shift}}\times \left(t=2020\right))\\ \times \left(\mathrm{Ki}H_{n,t}>0\right) \end{array}$$

where

*asc* = alternative specific constant,

*CD* = commuting distance,

*CHH* = couple household,

*KiH* = children under 10 years old in the household, and

*SHH* = single household.

The probability of person *n* choosing to WFH at period *t* is estimated as in the previous model (see formulas [3] and [4]).

#### Multiple Linear Regression Model on the Difference in Commuting Trips Between 2019 and 2020

To analyze the effects of COVID-19 on commuting behavior, a multiple linear regression model on the difference in the number of commuting trips between 2019 and 2020 was estimated. The difference in the number of trips is the dependent variable in the model. The model was estimated in SAS 9.4 (*43*). Participants with missing values or who reported anomalies in either year or changed work location between the two reports were excluded to control for reasons other than the pandemic influencing the number of commuting trips. The remaining sample size for the following analysis is 563. In this analysis, sociodemographic characteristics of the participants were included to assess who reduced the number of trips to work during the COVID-19 pandemic. Also, characteristics of the commuting trips were included. After running several models, the final one was selected based on the significance of the independent variables as well as the R-square. Including interaction effects did not improve the model; however, a variable was included to account for the different TGs that significantly influenced the model’s outcome. The final model accounts for the total CD in 2019, TGs, if the respondents commute by PT or car, and the age of the respondents. The estimated model can be expressed as:

(5) $$\begin{array}{c} V_{n}=\beta_{0}+\beta_{\mathrm{commute}}\times CD_{n}+\beta_{\mathrm{TG}}\times TG_{n}+\beta_{\mathrm{PT}}\times PT_{n}\\ +\beta_{\mathrm{car}}\times \mathrm{ca}r_{n}+\beta_{\mathrm{age}}\times \mathrm{ag}e_{n} \end{array}$$

where *CD* = commuting distance,

*TG* = telecommuting group, and

*PT* = public transport.

## Results and Discussion

This section presents and discusses the results of the analyses whereby all participants are first reported on and, subsequently, the different TGs are focused on.

### Changes in Possibility and Choice to Telecommute

**Figure 2.**
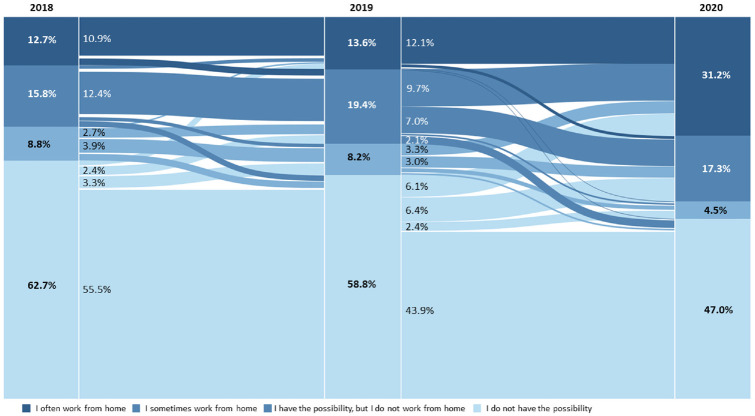
Change of work from home (WFH) status between 2018, 2019, and 2020 of respondents participating in all 3 years (*N* = 330).

As MOP makes it possible to analyze participants who reported in three subsequent years, it is possible to analyze the change in the WFH status from 2018 to 2019 compared with the change from 2019 to 2020. This allows for an analysis of the changes in telecommuting in years unaffected by the COVID-19 pandemic. Figure 2 illustrates the changes of telecommuting status in 3 years of the same participants. From the graph, it can be seen that the rate of telecommuters between 2018 and 2019 already increased. However, in 2020 this increase is much stronger. In 2018, 28.5% of respondents participated in telework. Few participants decreased the frequency of telework, and in 2019, 33% participated in telework. This indicates that there was already a trend toward telecommuting which was increased by the pandemic. The rate of those who chose not to telecommute even though they had the possibility stayed around the same between 2018 and 2019, whereas especially the number of occasional teleworkers increased in 2019.

**Figure 3.**
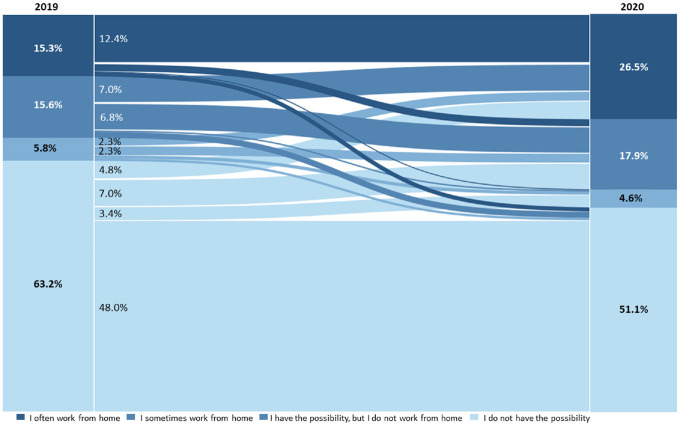
Change in work from home (WFH) status between 2019 and 2020 of respondents participating in both years (*N* = 789).

It has to be noted that people participating for three consecutive years are rather motivated and are often better educated, as described in the previous section, and usually drop out less frequently than less-educated respondents. As telecommuting is associated with higher degrees of education because of the nature of the respective jobs, there is a potential bias that increases with each additional year considered. To decrease the effect of this selection bias, only those participants who participated in 2019 and 2020, respectively, have been further analyzed. The changes in telecommuting status between these years is presented in Figure 3.

From the graph, it is possible to see an increase in people who WFH, and thus a decrease of people who do not telework. The share of people who WFH at least once a week increased from 15.3% in 2019 to 26.5% in 2020. Of those who occasionally telecommuted in 2019, 7% used this possibility more frequently in 2020. A considerable share of participants was also given the opportunity to telework in 2020. Of the respondents, 15.2% could not WFH in 2019 but could in 2020. From this data, it is possible to see a considerable share of respondents who never worked from home before the pandemic with an overall share of 16.4% of new telecommuters.

To analyze if the increased opportunity to telecommute was granted to people with different sociodemographic profiles, the results of a multinomial logit model are presented (Table 2). The model statistics show that, while the final model is better than the model only considering the ASC, the rho-squared value is relatively low. This is because of the limited available variables that influence the possibility to WFH. This is a choice that is not determined by the employees (i.e., the respondents) but the by the employers or the type of job. Thus, this model is solely based on sociodemographic data as proxy variables for the types of job.

**Table 2.** Variables and Results of the Logit Model on the Possibility to Work from Home (WFH)

Parameter	Attribute levels	Parameter value	Robust t-ratio
Alternative specific constant (ASC)	WFH possible	−1.1185	−6.27***
	WFH not possible *(const.)*	na	na
Shift ASC	WFH possible	0.5337	4.78***
	WFH not possible *(const.)*	na	na
Gender	Male *(const.)*	na	na
	Female	−0.3778	−2.79***
Highest degree of education	No degree *(const.)*	na	na
	High school diploma	0.9795	4.88***
	College or university degree	1.7012	10.77***
Economic status (ECS) of household	Low	−0.9343	−3.20***
	Medium	−0.7013	−4.64***
	High *(const.)*	na	na
Shift ECS of household	Low	−0.4276	−1.33*
	Medium	0.3208	1.84**
	High *(const.)*	na	na

*Note*: Observations 2,148; LL(start)/LL(0) −1,488.88; LL(final) −1,224.638; Rho-square 0.1775. Significance at the ***1%, **10%, *20% level. na = not applicable.

The estimates show that women as well as lower-educated people are less likely to be able to WFH. People with no degree (or lower high school degrees) are less likely to WFH than people who have a high school diploma at International Standard Classification of Education (ISCED) level 3. This relationship is even stronger for those who have a college or university degree. The estimates also show that the lower the ECS the less likely people are able to WFH. This is not surprising, as professional jobs are also linked to higher income. This has not been changed by the COVID-19 pandemic for respondents with a low ECS, on the contrary, the effects were even more severe as indicated by the negative shift. However, for respondents with a medium ECS, the chance to telecommute increased compared with respondents with a high ECS. The only other statistically significant shift effect identified was the shift of the ASC. The positive shift shows that, because of the COVID-19 pandemic, more people were given a chance to WFH, but these are not reflected in the sociodemographic variables of the model. This indicates that there was almost no shift in sociodemographic profiles of teleworkers and that telecommuting remains a privilege of male professionals with a high ECS as shown in studies conducted before the pandemic (*18*, *22*). The slight shift in the medium ECS support the findings presented by Kramer and Kramer that the possibility to telework was extended to a few other occupational groups (*31*). However, the results of this study show that the slight positive effect is dampened by the pandemic increasing the inequity of telecommuting possibility even further for low-income employees.

In the next model, the choice to WFH was analyzed. The results of the estimated logit model are presented in Table 3. The overall model fits are good, with an increased log-likelihood of the final model compared with the model at constant only and a relatively high rho-squared value.

**Table 3.** Variables and Results of the Logit Model on the Choice to Work from Home (WFH)

Parameter	Attribute levels	Parameter value	Robust t-ratio
Alternative specific constant (ASC)	WFH	1.5002	6.29***
	Not WFH *(const.)*	na	na
Shift ASC	WFH	0.6686	2.67***
	Not WFH *(const.)*	na	na
Commuting distance (CD)	Kilometers	0.0175	2.59***
Missing CD	No *(const.)*	na	na
	Yes	4.0032	4.04***
Shift children under 10 years old in the household (KiH)	No *(const.)*	na	na
	Yes	1.7854	1.71**
Single household (SHH)	Household (HH)-size > 1 *(const.)*	na	na
	HH-size = 1	–1.6525	–4.32**
Shift SHH	HH-size > 1 *(const.)*	na	na
	HH-size = 1	1.0128	1.90**
Couple household (CHH)	HH-size ≠ 2 *(const.)*	na	na
	HH-size = 2 (adults)	−0.7486	−2.74***

*Note*: Observations 2,148; LL(start)/LL(0) −594.0271; LL (final) −296.0819; Rho-square 0.5016. Significance at the ***1%, **10% level. na = not applicable.

The parameter estimates show that CD positively influences the choice to WFH; that is, respondents with longer commutes are more likely to WFH. The parameter itself is relatively small compared with the other parameters, which is attributed to the scale of CD compared with the other variables, which are all dummy coded. Interestingly, it was not possible to identify a statistically significant shift parameter for CD. This indicates that the influence of CD was not suspended during the pandemic and that, generally speaking, people who live closer to their workplace commuted more often than those with a longer commute, even during the pandemic. This finding is consistent with those presented by Schneider and Schinkowsky who find that, during the pandemic, telecommuters living closer to their workplace evaluate telecommuting less positively than those living further away (*32*).

All other variables in the model factor in the household configuration. The parameter for a household consisting of a couple of adults without children is negative, indicating that the presence of a partner in the household discourages people from WFH. Although the survey did not gather information on the respondents’ living conditions, this effect suggests that telework can put a strain on the relationship, as found in previous studies (*44*). There was also no significant shift parameter for this variable, indicating that this pressure on two-person households was not ignored during the pandemic and employees living with a partner tended to still choose to commute to work rather than telecommute. People living alone also tend to choose not to telecommute, probably because the workplace is a source of social interaction (*45*, *46*). However, for this variable, a positive shift parameter was identified. During the pandemic, the risk of infection may have been perceived higher than the feeling of missing social interaction. Furthermore, although the pandemic increased social isolation, it can be assumed that WFH policies of a company would also affect the colleagues of respondents living alone, thus they would not have increased social interaction even if they went to work on-site. Therefore, WFH may not have decreased social interaction because there would not have been any social interaction in the workplace either. It can be assumed that this group of people will not tend to WFH as social interactions in the workplace increase in a post-pandemic situation.

The parameter for KiH is was, surprisingly, not significant. However, the shift parameter indicated that childcare responsibilities were perceived differently during the pandemic. The positive parameter suggests that people who have children under the age of ten were more likely to telecommute during the pandemic. Respondents who have child care responsibilities benefit from WFH, which was especially helpful during the pandemic, as schools and kindergartens had partially closed or classes were put under quarantine regularly.

### Differences Between Non-Telecommuters, New Telecommuters and Experienced Telecommuters

In this part of the results section, the differences in telecommuting experience are looked at, and non-telecommuters, new telecommuters, and experienced telecommuters are distinguished.

**Figure 4.**
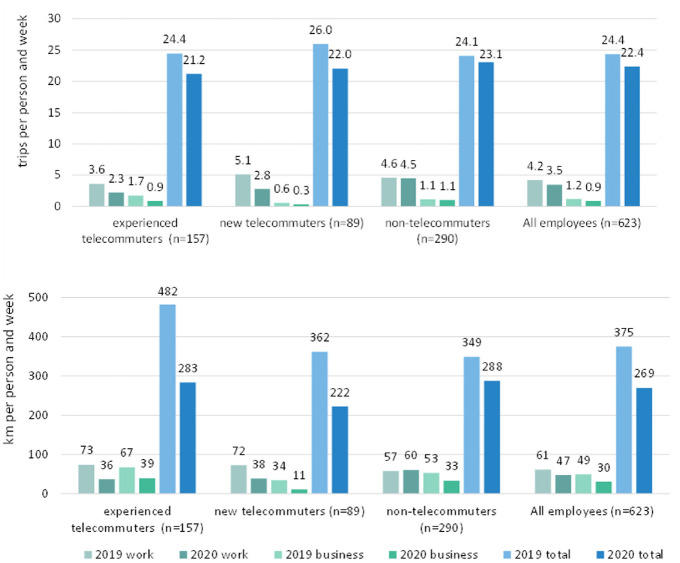
Trips made (*top*) and distances traveled (*bottom*) to work and for business and all trip purposes, differentiated by telecommuting group (TG) in 2019 and 2020.

The increased use of WFH because of the pandemic has affected the transport system considerably, as mobility, and especially commuting, is reduced. To this aim, the trip diary data of MOP is analyzed. First, the reduction of trips and distances traveled in 2020 compared with the previous year are identified (Figure 4). Furthermore, paired-sample t-tests were performed at 95% confidence level on both samples to examine differences between the trips made and distances traveled for work, business trips, and the all trips.

As shown in Figure 4, the total number of trips and person kilometers traveled (PKT) for working and business purposes as well as all trips (total) within one week dropped considerably between 2019 and 2020. The analyses of work and business trips only consider one direction of the trips, whereas the calculation of trips and distance totals considers both directions of the respective trips. On average, employees made 24.4 trips per week in 2019 and 22.4 trips per week in 2020. Based on the t-test, a significant decrease (t = 7.86, *p < *0.001) of 28% can be seen between these years in relation to PKT. From the graph it can be seen that experienced telecommuters show higher PKT under non-pandemic conditions than new telecommuters and non-telecommuters. This applies to work and business travel and the overall figures.

Experienced telecommuters already made fewer trips to work in 2019 (3.6 trips per week) than new telecommuters (5.1 trips per week) and non-telecommuters (4.6 trips per week). This pattern can also be observed in 2020. Experienced telecommuters also reduced their small number of trips to work significantly (t = 6.40, *p < *.0001), as did new telecommuters (t = 6.49, *p < *.0001). Non-telecommuters show no significant changes in work and business trips. Business trips were significantly reduced by experienced telecommuters (t = 3.55, *p < *0.0005) and new telecommuters (t = 2.11, *p < *0.030). A possible explanation for the reduction of business trips for experienced and new telecommuters is that business trips were replaced by digital services, as well as cancelled service contracts that would have resulted in a business-related trip. Overall, experienced telecommuters made 1.7 business trips per week in 2019, new telecommuters made 0.6 business trips per week, and non-telecommuters made 1.1 business trips per week. The differences indicate that experienced telecommuters’ jobs include working in different locations and that business travel is also an essential part of their job.

For all employees, PKT decreased significantly between 2019 (375 km/week) and 2020 (269 km/week) (t = 7.86, *p* < .001). Thereby, the traveled distances in 2020 differed significantly from those in 2019. Although experienced telecommuters made fewer work-related trips than new telecommuters and non-telecommuters in 2019, they present with a considerably higher value of PKT (482 km/week) compared with new telecommuters (362 km/week) and non-telecommuters (349 km/week). This means that experienced telecommuters spend a large part of their weekly mileage on non-work-related trips. These results support earlier findings indicating that WFH does not decrease travel demand of teleworkers (*12* [Bibr bibr13-03611981221089938][Bibr bibr14-03611981221089938]–*15*). With COVID-19-related closures of recreational facilities, a major source for activity generation was eliminated, resulting in a significant decrease (t = 5.49, *p* < .001) in PKT to 283 km/week in 2020.

New telecommuters also show significant decreases in PKT (t = 3.65, *p* < .001), but also on work trips (t = 4.23, *p* < .001) as a result of WFH. Non-telecommuters also show a significant decrease in total distances traveled (t = 3.64, *p* < .001). Since this group could not or would not switch to telework, no significant changes in relation to work-related transport performance can be seen. This means that these respondents also mainly limited their leisure trips.

**Figure 5.**
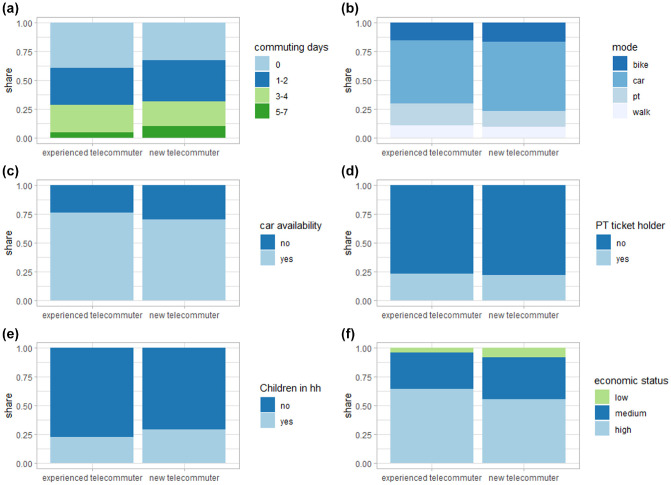
Experienced versus new telecommuters in 2020 by: (*a*) number of days with at least one commuting trip, (*b*) modal split for commuting trips, (*c*) car availability, (*d*) public transport (PT) ticket ownership, (*e*) number of children in the household (KiH), and (*f*) economic status (ECS).

A closer comparison of new and experienced telecommuters reveals that there are differences between them concerning their commuting behavior and sociodemographic profiles, as shown in Figure 5. From the graph it can be seen that both groups include a considerable share of people who did not commute to work any day of the week; however, the effect is higher for experienced telecommuters. A reverse effect can be identified for the category of 5–7 commuting days. New telecommuters make up a larger share than experienced telecommuters for this group. The two groups show almost no differences in mode choice behavior on commuting trips. The majority of respondents chose to drive to work with a slight shift toward PT for experienced telecommuters. This suggests that few (if any) adjustments have to be made to mode choice models and that using data gathered during the pandemic for commuting mode choice models as suggested by Hensher et al. is sensible (*33*). Similarly, no considerable difference can be identified concerning PT season ticket ownership. A slight difference can be identified concerning car availability. Over 75% of experienced telecommuters have access to a car. Comparing these rates with the modal splits on commutes, the results are consistent with those presented by e Silva and Melo indicating that telecommuters use more polluting modes on non-work trips (*13*).

Considering the presence of children in the household, compared with experienced telecommuters, a larger share of new telecommuters have at least one child in the household. This supports the shift parameter in the previous logit model on the choice to telecommute and suggests that a decrease of WFH from new telecommuters can be expected as regular child care resources become available again. Furthermore, the ECS of the two groups show different distributions. A larger share of telecommuters has a low or medium ECS compared with experienced telecommuters. This also supports previous findings from the logit model and literature (*31*).

**Figure 6.**
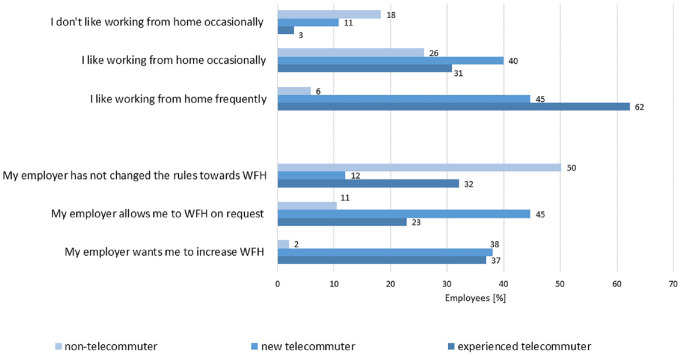
Perception of working from home (WFH) by telecommuting group (TG).

In the MOP survey, participants were also asked how they perceive WFH and what their employers do to facilitate telework to increase social distancing. Figure 6 shows how experienced telecommuters, new telecommuters, and non-telecommuters perceive WFH and what they think their employers expect of them. Experienced telecommuters (62%) have a positive perception of frequent telecommuting. In contrast, only 2% of non-telecommuters would like to frequently WFH, and 11% of them would like to occasionally WFH. Unsurprisingly, the vast majority of those who do not like WFH are non-telecommuters. It should be noted that this question is prone to selection bias. Of the non-telecommuters, 50% did not agree with any of the first three statements about telework. This response behavior can be explained by the lack of experience of WFH. Those who did not answer these questions are most likely respondents whose occupation or employer (or both) do not allow for telework and thus these questions are not applicable to them.

Interestingly, the experienced (37%) and new (38%) telecommuters report that their employer wants them to increase WFH, whereas non-telecommuters do not tend to report this (2%). Instead, non-telecommuters report that their employer has not made any changes (50%).

To infer further insights into who reduced their number of commuting trips in 2020 compared with 2019, a multiple linear regression model was estimated. The results are given in Table 4. In this model, interaction effects were also tested for. In this case, it was assessed if the telecommuting status shifted the relationship between any of the independent variables and the dependent variables. However, no such effect was identified. Thus, only the TGs were included as regressors in the model.

**Table 4.** Results of Multiple Linear Regression Model on the Change in Trips to Work between 2019 and 2020

Parameter	Estimate (*β*)	Standardized estimate (B)	Standard error	t-value	Pr > |t|
Intercept	1.9740	0.0000	0.3460	5.71	<.0001
Total commuting distance (CD) in 2019	−0.0095	−0.2875	0.0013	−7.29	<.0001
Telecommuting group (TG): experienced telecommuter	−0.5895	−0.1114	0.2172	−2.71	0.0069
TG: new telecommuter	−1.8604	−0.2812	0.2620	−7.10	<.0001
TG: non-telecommuter	0.0000	0.000	na	na	na
Non-public-transport (PT)-commuters	−0.9500	−0.1311	0.2862	−3.32	0.0010
PT-commuters	0.0000	0.0000	na	na	na
Non-car-commuters	−1.3936	−0.2864	0.2103	−6.63	<.0001
Car-commuters	0.0000	0.0000	na	na	na
Younger than 55 years	−0.3085	−0.0628	0.1829	−1.69	0.0922
55 years old or older	0.0000	0.0000	na	na	na

*Note*: Observations *N* = 563; R-Square 0.2317; F value 27.95; Pr > F < .0001. na = not applicable.

The overall model is significant (<.0001). The R-square is low (0.2317), which means that the model only explains a little of the variability. The inclusion of additional characteristics did not lead to a higher R-square. Other sociodemographic characteristics—gender, age, children in household, ECS, education level, and household size—did not improve the model. No significant relationships to the change in number of trips to work were identified for them. This indicates that the reduction of trips to work is rather independent of the household composition and the sociodemographic characteristics. Overall, the experience with telecommuting has a highly significant effect on the change in the number of trips as well as the characteristics of commuting (total CD in 2019 and commuting mode).

A negative estimate indicates that the number of trips to work in 2020 was reduced compared with 2019. When interpreting the results, it has to be considered that there was an overall decrease in the number of trips to work, as already shown in Figure 4. The total CD in 2019 describes the sum of distances traveled to work in the reported week in 2019. The negative parameter means that the higher the sum of distances traveled to work in 2019, the more likely people were to reduce the number of trips to work in 2020. This result is indicative of an increased tendency to telecommute more often with longer CDs, confirming findings of an earlier study on telecommuting frequency and CD by Mokhtarian et al. (*47*). In addition, people not commuting by car were more likely to reduce the number of trips. People commuting by car may have felt more comfortable traveling in their own private car, while people who traveled by other modes were more likely to reduce their number of trips to work. Surprisingly, the same holds true for PT, albeit with a weaker effect. One possible explanation for this is that people who commute by PT may have had less opportunity to WFH and are dependent on going to work even if that meant they had to increase their risk of infection by traveling by PT. However, this parameter value was unanticipated and additional data is required to further analyze the underlying effects.

The parameters for new telecommuters and experienced telecommuters are both negative, as expected, as they are very likely to reduce the number of trips compared with 2019. The value for new telecommuters is much lower compared with the experienced telecommuters. This does not mean that new telecommuters made fewer trips than experienced telecommuters, but that their commuting frequency decreased more compared with the year before. This shows that new telecommuters made bigger adjustments to their daily rhythms compared with experienced telecommuters. For new telecommuters, the pandemic can be considered a turning point and possibly a permanent break in their habits. Employed people, especially, show stable activity patterns over time, and habitual travel patterns are hard to break out of without external pressure (*48* [Bibr bibr49-03611981221089938]–*50*).

The participant’s age was the only one of several sociodemographic variables tested which shows a significant relationship to the dependent variable. People younger than 55 years were more likely to reduce the number of trips to work between the years. Several simulations were run with varying configurations of the age parameter. Neither a linear consideration nor different age categories provided statistically significant results. There are several reasons why categorizing people into the groups younger than 55 years and 55 years or older resulted in significant findings: older employees are usually less tech-savvy leading them to not adjust to WFH as well as their younger counterparts. Also, people in this age group are more likely to hold managerial positions and, thus, telecommuted less frequently, as management of fully virtual teams is more challenging (*45*). And, lastly, people older than 55 years are far less likely to have smaller children and were therefore not affected by school closings.

### Implications

The results of this study have implications both for the time of the ongoing pandemic (and future pandemics) and a post-COVID-19 period.

#### Implications Concerning the Period During the Pandemic

Although a slight shift of the ECS of telecommuters was identified, there is still a large disparity between the three different groups. This leads to a discussion of social equity, since a gap between the higher and lower ECS becomes prevalent. People with a high ECS are usually in a position of great autonomy and earning a high salary which makes them more likely to have the possibility to WFH, and thus the risk of infection can be reduced by staying at home while earning the same as before. In contrast, people working in manufacturing, for example, who usually have lower salaries, have no possibility for telecommuting and thus have a higher risk of infection or have to take a leave of absence or even quit their job if they want to reduce the risk of infection. This effect has been described by Shakibaei et al. The results of their study show that the high fuel costs in Turkey forced people to use PT for commuting during the pandemic even if they owned a car (*51*). Policymakers should draw two conclusions from this. First, the possibility of WFH should be introduced wherever possible. An example of how to achieve this is that incentives could be given to employers to make it more worthwhile for them to offer WFH. Second, people who do not have jobs that allow them to telecommute should be offered the best possible protection against infection. For example, large PT capacities can help minimize exposure during commuting.

#### Post-Pandemic Implications

For the post-pandemic situation, increased use of WFH is seen as a measure to reduce traffic congestion in transportation. However, as this study shows, experienced telecommuters presented with higher PKT than new and non-telecommuters before the pandemic and are likely to pick up as soon as the pandemic is over. Furthermore, in light of the existing disparity with regard to who is able to WFH, policies that aim to increase telecommuting should be carefully chosen. Raising the costs for commuting as a measure to promote telecommuting are likely to hurt those who are already in jobs with lower income and who cannot WFH (*22*, *52*). Therefore, it is suggested, rather, that sustainable mode choice behavior is supported for (infrequent) commuting trips of teleworkers; for example, by offering PT tickets as employer benefits which often become less profitable for telecommuters as they reduce the frequency of their commutes.

It also remains to be seen how many new telecommuters will continue to WFH after the pandemic. Although most have a positive perception of telework, people living alone will likely return to commute to work or telecommute less frequently as working at the office should also be seen as a place for social contact. The results suggest that this will also hold true for people in households with smaller children with the re-opening of schools. Furthermore, new teleworkers became familiar with WFH under peculiar conditions in which entire teams were managed virtually and worked remotely. Therefore, questions about the perception of telecommuting should be restated after the effects of the pandemic have subsided. It is foreseeable that there will also be hybrid remote working solutions in the future, in which both employers and employees benefit from the possibility of WFH, but still retain the benefits of working on-site from time to time. This may change opinions on WFH, especially among new telecommuters.

Furthermore, the results of this study suggest that transportation modelers wanting to integrate telecommuting behavior based on data collected during the pandemic should take the differences of experienced and new telecommuters into account. The suggestion is also made to generate models with a high level of detail about sociodemographic and, first and foremost, occupational information.

## Conclusions

This paper analyzes the effects of the COVID-19 pandemic on telecommuting behavior using travel survey data in Germany. With the panel design, MOP provides unique data of individuals who participated before and during the pandemic. Thus, the data allows for the evaluation of changes in behavior triggered by the pandemic and differences between experienced, new, and non-telecommuters. These changes were analyzed and evaluated applying descriptive methods, and linear and logistic regression.

Generally, the sociodemographic profiles of new telecommuters show only slight differences compared with experienced teleworkers. Experienced telecommuters tend to have a high ECS compared with new telecommuters. A positive shift parameter was further identified for the medium ECS compared with a high ECS influencing the possibility to WFH. Together, these findings indicate that, as a result of the pandemic, new types of occupation were conducted remotely.

Concerning commuting behavior before and during the pandemic, the results show that PKT and number of trips during the pandemic were low for all three groups. However, experienced telecommuters presented with high PKT values before the pandemic, while new and non-telecommuters showed values close to the mean of the entire employed population.

The results of this study further suggest that the transition induced by the pandemic was more drastic for new telecommuters compared with experienced telecommuters.

The choice to telecommute is influenced by CD with no shift effect induced by the pandemic. The COVID-19 pandemic had an effect on how household configurations are considered in the choice to WFH, as a positive shift of children in the household was identified. Also, while people living alone are less likely to choose to telecommute, this effect was reduced as a result of the pandemic.

While it was possible to capture diverse aspects of telecommuting and the effects on commuting behavior, there are also shortcomings of the study. The data used comes from a panel survey on travel behavior conducted for more than 25 years in the same design without major changes. Therefore, the survey is not explicitly designed to capture WFH. The information on WFH is only available in broad categories and no details on the type of occupation (office, industry, etc.) are captured.

Furthermore, only the view of employees is captured in the data and not that of employers. Concerning the sample characteristics, the share of high-educated people is higher than in the overall population. While this is good for the focus of this study on people who use WFH, it does not allow for extrapolation to the population as a whole.

The study is based on data from Germany and transferability is limited. Although many of the presented results are consistent with previously presented studies from other countries, the behavior of respondents during the 2020 survey period was influenced by policy measures which differed from country to country.

Future work will include the transferal of these analyses to other longitudinal data to see how the results compare with international contexts and to evaluate if especially the possibility to telecommute can be modeled better. Additionally, as the 2021 survey wave was conducted in a period with relatively low infection rates, it will be possible to evaluate how the new telecommuters adjusted their behavior in low-risk times. These analyses will be conducted once the data are available.
